# Supplementary material for: The impact of the COVID-19 pandemic on microbial keratitis presentation patterns
Source: PLoS One. 2021 Aug 18;16(8):e0256240. doi: 10.1371/journal.pone.0256240 (PMC8372897; doi:10.1371/journal.pone.0256240)
Supplement: S1 Table — (DOCX) [file pone.0256240.s001.docx]

| **S1 Table. Admissions to the eye ward activity 2015-2016** | | | | |
| --- | --- | --- | --- | --- |
|  | **Ambulatory/Day Case** | **Elective** | **Emergency** | **Total** |
| **Patients (n)** | 255 | 491 | 449 | 1195 |
