# Supplementary material for: The impact of the COVID-19 pandemic on microbial keratitis presentation patterns
Source: PLoS One. 2021 Aug 18;16(8):e0256240. doi: 10.1371/journal.pone.0256240 (PMC8372897; doi:10.1371/journal.pone.0256240)
Supplement: S2 Table — (DOCX) [file pone.0256240.s002.docx]

| **S2 Table. Complete list of risk factors** | | |
| --- | --- | --- |
| **Active ocular surface disease (OSD)** | **Previous keratitis** | **Previous Surgery/Trauma** |
| Conjunctivitis | Keratitis (Viral) | Corneal Crosslinking |
| Corneal Anaesthesia (Neurotropic) | Keratitis (Bacterial) | Corneal Transplant (previous) |
| Corneal Decompensation | Keratitis (Fungal) | Corneal Trauma (old) |
| External Disease: Acne Rosacea) | Keratitis (infectious, non-specified) | Laser Refractive Surgery |
| External Disease: Anterior Lid | Marginal keratitis | Ocular Surgery (>6 month) |
| External Disease: Atopy |  | Ocular Surgery Recent (<= 6 month) |
| External Disease: Ectropion |  |  |
| External Disease: Entropion |  |  |
| External Disease: Floppy Eye Lid Syndrome |  |  |
| External Disease: Generalised BKC |  |  |
| External Disease: Immune OSD |  |  |
| External Disease: Posterior Lid |  |  |
| External Disease: Toxic OSD (e.g. drop related)) |  |  |
| External Disease: Trichiasis |  |  |
| Keratitis - Viral (Active) |  |  |
| Keratitis (Acanthamoeba) |  |  |
| Keratoconus |  |  |
| Marginal keratitis (active) |  |  |
| Scleritis |  |  |
