# Supplementary material for: The impact of the COVID-19 pandemic on microbial keratitis presentation patterns
Source: PLoS One. 2021 Aug 18;16(8):e0256240. doi: 10.1371/journal.pone.0256240 (PMC8372897; doi:10.1371/journal.pone.0256240)
Supplement: S3 Table — (DOCX) [file pone.0256240.s003.docx]

| **S3 Table. Trends in patient characteristics across the pre-C19 period** | | | | | |
| --- | --- | --- | --- | --- | --- |
|  | **2017** | **2018** | **2019** | **p-Value**  **2017-19** | **2020** |
| Total number of admissions (n) | ≈ 10,000 § | 12,128 | 12,239 | - | 5,759 |
| Patients with MK (n) | 63 | 50 (0.4%) | 68 (0.6%) | 0.117 § | 49 (0.9%) |
| Age (years) | 55.3 ± 21.9 | 56.6 ± 21.3 | 54.8 ± 20.5 | 0.922 | 53.3 ± 17.8 |
| Sex – male (%) | 34 (54.0%) | 27 (54.0%) | 34 (50.0%) | 0.879 | 31 (63.3%) |
| Ethnicity |  |  |  | 0.299 |  |
| *White* | 46 (75.4%) | 32 (71.1%) | 34 (58.6%) |  | 27 (71.1%) |
| *Asian* | 11 (18.0%) | 10 (22.2%) | 17 (29.3%) |  | 7 (18.4%) |
| *Black* | 2 (3.3%) | 1 (2.2%) | 6 (10.3%) |  | 3 (7.9%) |
| *Mixed / Other* | 2 (3.3%) | 2 (4.4%) | 1 (1.7%) |  | 1 (2.6%) |
| IMD decile |  |  |  | 0.238* |  |
| *1-3* | 31 (50.0%) | 26 (52.0%) | 39 (59.1%) |  | 26 (53.1%) |
| *4-7* | 16 (25.8%) | 20 (40.0%) | 22 (33.3%) |  | 19 (38.8%) |
| *8-10* | 15 (24.2%) | 4 (8.0%) | 5 (7.6%) |  | 4 (8.2%) |
| Laterality – right (%) | 32 (50.8%) | 28 (56.0%) | 29 (42.6%) | 0.351 | 27 (55.1%) |
| Duration of symptoms (Days) | 3 (1-6) | 3 (1-7) | 3 (2-5) | 0.965 | 4 (2-7) |
| **Risk factors** | | | | | |
| Contact lens | 20 (31.7%) | 20 (40.0%) | 20 (29.4%) | 0.476 | 14 (28.6%) |
| Underlying OSD (active)** | 31 (49.2%) | 17 (34.0%) | 32 (47.1%) | 0.217 | 26 (53.1%) |
| Previous keratitis*** | 10 (15.9%) | 4 (8.0%) | 6 (8.8%) | 0.362 | 7 (14.3%) |
| Previous ocular surgery/trauma | 12 (19.0%) | 7 (14.0%) | 12 (17.6%) | 0.801 | 10 (20.4%) |
| Concurrent trauma | 6 (9.5%) | 0 (0.0%) | 4 (5.9%) | 0.064 | 8 (16.3%) |
| Corneal foreign body | 1 (1.6%) | 0 (0.0%) | 2 (2.9%) | 0.780 | 2 (4.1%) |
| Diabetes mellitus | 6 (9.5%) | 8 (16.0%) | 3 (4.4%) | 0.110 | 7 (14.3%) |
| Rheumatoid arthritis | 2 (3.2%) | 1 (2.0%) | 3 (4.4%) | 0.879 | 1 (2.0%) |
| Systemic immunosuppression | 2 (3.2%) | 1 (2.0%) | 0 (0.0%) | 0.378 | 6 (12.2%) |
| Thyroid eye disease | 0 (0.0%) | 1 (2.0%) | 0 (0.0%) | 0.276 | 0 (0.0%) |

*Abbreviations: OSD, ocular surface disease; IMD, index of multiple deprivation; MK, microbial keratitis). Continuous variables are reported as mean ± SD or median (interquartile range), with p-values from Kruskal-Wallis tests. Categorical variables are reported as N (column %), with p-values from Fisher’s exact tests, unless stated otherwise. All p-values represent comparisons across the three Pre-C19 years (2017, 2018 and 2019), and bold p-values are significant at p<0.05. Data from the year 2020 are also reported, for reference. For risk factors “previous” denotes that the risk factor had healed prior to onset of MK.*

**p-Value from Kruskal-Wallis test, as the factor is ordinal.*

***Ocular surface disease, such as dry eye, affecting the patient at the time of presentation – a full list of included diseases is reported in Supplementary Table 1.*

****Viral/bacterial/fungal/parasitic/marginal disease.*

*§The figure for 2017 is an estimated figure, as this was transitional year for departmental electronic patient management systems, resulting in inaccurate recording of patient activity. Since the actual figure is not available, an estimate of activity is included. The p-value comparing the proportions of patients with MK excludes 2017; hence represents a comparison of 2018 vs. 2019.*
