# Supplementary material for: The impact of the COVID-19 pandemic on microbial keratitis presentation patterns
Source: PLoS One. 2021 Aug 18;16(8):e0256240. doi: 10.1371/journal.pone.0256240 (PMC8372897; doi:10.1371/journal.pone.0256240)
Supplement: S4 Table — (DOCX) [file pone.0256240.s004.docx]

| **S4 Table. Causes of concurrent ocular trauma** | |
| --- | --- |
| **Pre-C19** | **Y2020** |
| Garden related x 4 | Finger x3 |
| Work related x 3 | Not specified x 2 |
| Finger x 2 | Fist (assault) x1 |
| Eye dropper x 1 | Wall/pillar x1 |
|  | Elastic band from mask x 1 |

*Abbreviations: Pre-C19, Pre-COVID-19 Years (2017,2018,2019); Y2020, year 2020*
